# Assessment of the Costs Related to West Nile Virus Monitoring in Lombardy Region (Italy) between 2014 and 2018

**DOI:** 10.3390/ijerph19095541

**Published:** 2022-05-03

**Authors:** Francesco Defilippo, Michele Dottori, Davide Lelli, Mario Chiari, Danilo Cereda, Marco Farioli, Rosa Chianese, Monica Pierangela Cerioli, Francesca Faccin, Sabrina Canziani, Tiziana Trogu, Enrica Sozzi, Ana Moreno, Antonio Lavazza, Umberto Restelli

**Affiliations:** 1 Istituto Zooprofilattico Sperimentale della Lombardia e Emilia-Romagna, Via Bianchi 7/9, 25124 Brescia, Italy; michele.dottori@izsler.it (M.D.); davide.lelli@izsler.it (D.L.); monicapierangela.cerioli@izsler.it (M.P.C.); francesca.faccin@izsler.it (F.F.); sabrina.canziani@izsler.it (S.C.); tiziana.trogu@izsler.it (T.T.); enrica.sozzi@izsler.it (E.S.); anamaria.morenomartin@izsler.it (A.M.); antonio.lavazza@izsler.it (A.L.); 2 DG Welfare, Regione Lombardia, Piazza Città di Lombardia 1, 20124 Milan, Italy; mario_chiari@regione.lombardia.it (M.C.); danilo_cereda@regione.lombardia.it (D.C.); marco_farioli@regione.lombardia.it (M.F.); 3 SRC Lombardia-Struttura Regionale di Coordinamento per le Attività Trasfusionali della Lombardia, Agenzia Regionale Emergenza Urgenza, Via A. Campanini, 6, 20124 Milano, Italy; r.chianese@areu.lombardia.it; 4 Dipartimento Interaziendale di Medicina Trasfusionale ed Ematologia e Servizio di Immunoematologia e Medicina Trasfusionale, ASST dei Sette Laghi, Viale L. Borri, 57, 21100 Varese, Italy; 5 Center for Health Economics, Social and Health Care Management, LIUC-Università Cattaneo, Corso Matteotti 22, 21053 Castellanza, Italy; urestelli@liuc.it; 6 School of Public Health, Faculty of Health Sciences, University of the Witwatersrand, 27 St Andrews Road, Parktown, Johannesburg 2193, South Africa

**Keywords:** blood donors, surveillance, mosquitoes, human, horses, NAT

## Abstract

In Italy, the West Nile Virus surveillance plan considers a multidisciplinary approach to identify the presence of the virus in the environment (entomological, ornithological, and equine surveillance) and to determine the risk of infections through potentially infected donors (blood and organ donors). The costs associated with the surveillance program for the Lombardy Region between 2014 and 2018 were estimated. The costs of the program were compared with a scenario in which the program was not implemented, requiring individual blood donation nucleic acid amplification tests (NAT) to detect the presence of WNV in human samples throughout the seasonal period of vector presence. Considering the five-year period, the application of the environmental/veterinary surveillance program allowed a reduction in costs incurred in the Lombardy Region of 7.7 million EUR. An integrated surveillance system, including birds, mosquito vectors, and dead-end hosts such as horses and humans, can prevent viral transmission to the human population, as well as anticipate the detection of WNV using NAT in blood and organ donors. The surveillance program within a One Health context has given the possibility to both document the expansion of the endemic area of WNV in northern Italy and avoid most of the NAT-related costs.

## 1. Introduction

West Nile Virus (WNV) is a flavivirus transmitted by ornithophilic mosquitoes between avian hosts [1]. Virus amplification within avian and mosquito populations may lead to spillover to incidental mammalian hosts. In particular, humans and horses are considered incidental dead-end hosts for WNV; in fact, they are not part of the virus transmission cycle because they do not produce viremia sufficient to infect mosquitoes [2]. In humans, the WNV infection is mostly asymptomatic, although 20% of cases may develop flu-like symptoms and around 1% may develop neuro-invasive symptoms, potentially lethal for elderly and immunosuppressed individuals [3]. In addition to the direct risk to human health, asymptomatic blood donors represent a recognized problem for the safety of blood transfusions in affected areas [4]. In fact, transmission of the WND infection through blood transfusion as well as organ transplantations has been demonstrated [5,6].

The ecologic aspects of WNV infection were first described in the 1950s in Egypt [7] and it is now considered the most widespread arbovirus in the world [8]. In Europe, the first WNV outbreak in humans was reported in 1962–1963 in France, but the virus has been circulating in Europe since the 1950s, as shown by serological surveys [9]. Annual fluctuations in WNV activity have been assessed by the number of cases reported in European Union/European Economic Area (EU/EEA) countries and EU neighboring countries: 3067 cases (2018), 477 cases (2019), and 322 cases (2020). The 2018 spike in European cases could have been due to the unusually hot summer [10,11].

In addition, the economic impact of WNV infections might be relevant for health services around the world. Two published analyses, related to the context of the United States, reported costs for hospitalization equal to 778 million USD (US Dollar) between 1999 and 2012, with median initial costs of hospitalization (both direct and indirect) per case between 7501 and 82,542 USD, and median long-term costs (both direct and indirect) between 7025 and 76,747 USD among patients with fever, meningitis, encephalitis, and acute flaccid paralysis [12,13].

In Italy, the first outbreak of WNV infection was reported in 1998 in the Tuscany region [14], and then the virus re-emerged after 10 years in August 2008 involving eight provinces in three regions (Emilia Romagna, Veneto, and Lombardy).

Due to the re-emergence and the following geographical spread of WNV, the Directorate General for Prevention of the Italian Ministry of Health (MoH) issued in the spring of 2010 a national plan for WNND human surveillance that integrated human and veterinary surveillance [15].

Since then, WNV has been repeatedly identified through integrated surveillance programs conducted in Italian regions [16], including Lombardy [17] and Emilia Romagna [18]. In addition, human cases of WNND have been reported every year in Italy [19].

The occurrence of the WNV infection cycle is favored by geographic aspects (low altitude, presence of rivers) and climate (high temperature in the warmest month, high annual temperature range) [20]. These characteristics are those commonly present in the northern Italian regions of the Po basin, such as Piedmont, Lombardy, Veneto, and Emilia-Romagna. In these regions, a specific surveillance program has been active for more than 15 years to assess the presence of West Nile Disease infections [21] and consists of two distinct but complementary activities: epidemiological and environmental surveillance [22].

Epidemiological surveillance identifies human cases to quantify the disease burden and to identify seasonal, geographical, and demographic patterns of human morbidity and mortality, while environmental/veterinary surveillance monitors local WNV activity in vectors and nonhuman vertebrate hosts in advance of epidemic activity affecting humans. The aim of the surveillance program is to address and to maximize the response of healthcare providers and professionals via the early detection of the virus in the different epidemiological actors, as well as focus the interventions toward target areas and seasonal periods. The WNV surveillance plan considers a multidisciplinary approach to identify the presence of the virus in the environment (entomological, ornithological, and equine surveillance) and to determine the risk of infections through potentially infected donors (blood and organ donors) [23].

According to the indications coming from the environmental/veterinary surveillance, the analyses conducted to detect the presence of WNV in blood samples for transfusions, as well as all activities related to organ donations, are conducted starting from the date of the first positive sample identified in mosquitoes, birds, or horses, instead of being performed for a fixed period from the beginning of June to the end of November. Such an integrated and multidisciplinary WNV surveillance system, which includes, in addition to humans, wild birds, mosquitoes and horses, has been implemented in Lombardy Region since 2014. The main aims of this surveillance system are (1) early detection of the circulation of WNV in the environment, and (2) mitigation of the risk of viral transmission through blood and organ donations [24]. The detection of WNV in one of the target species of the surveillance system in a given province has led to the start of a systematic individual blood donation nucleic acid amplification testing (NAT) program in that province until the end of the annual transmission season (end of November), as required by the European regulation [25].

This One Health approach is based on the collaboration of different public institutions, in a network that connects humans, animals, and environmental health, under the coordination of the working group of the Regional Health and Welfare Unit.

There have been few studies establishing the economic efficiency of One Health approaches to disease mitigation [26]. However, such information is critical for the development of cost-effective control of zoonoses, including WNV. Therefore, the objective of this study was to assess the costs related to WNV surveillance in Lombardy Region between 2014 and 2018 and to estimate the costs avoided, particularly related to NAT not performed, thanks to the environmental/veterinary surveillance, by adopting the point of view of the Regional Health and Welfare Unit.

## 2. Materials and Methods

The analysis was conducted considering the costs associated with the environmental/veterinary surveillance program, the direct medical costs associated with the screening activity for blood donors and organ donations, and the costs associated with the management of WNV infections including hospitalization.

Furthermore, the costs of the program were compared with a scenario in which the program was not implemented, requiring only NAT analyses to detect the presence of WNV in human samples throughout the possible transmission period. The costs considered in the analysis are referred to each reference year.

### 2.1. Environmental and Veterinary Surveillance

With the objective of assessing the presence in the environment of WNV between May and October, veterinary surveillance was conducted in three areas of activity, targeting different species: *Culex* mosquitoes (entomological surveillance), birds (ornithological surveillance), and horses (equine surveillance).

Entomological surveillance was performed through fortnightly sampling with attraction traps, between May and October. The number of traps progressively increased from 38 at the beginning (2014) to 44 in total in 2020. They were placed in areas suitable for circulation of the virus vector [27] and distributed regularly in the region so as to cover the whole territory divided into 20 × 20 km^2^ areas.

Ornithological surveillance was performed through active surveillance (capture and killing of resident “reservoir” birds of selected species) and through passive surveillance (identifying episodes of abnormal wildlife mortality or analyzing animal death in wildlife refuge center). Carcasses of magpie (*Pica pica*), hooded crow (*Corvus corone cornix*), and Eurasian jays (*Garrulus glandarius*) were collected and delivered to the laboratory by rangers and hunters. These species are considered pests for crops and, therefore, are under population control programs, authorized yearly by the National Institute for Wildlife (ISPRA). The sampling from passive surveillance mainly involved birds belonging to the orders Accipitriformes, Charadiiformes, Columbiformes, Falconiformes, and Passeriformes.

Equine surveillance was performed through clinical assessment of neurologic symptoms in horses.

Table 1 provides summarized information of all cost items considered in the analysis.

To determine the costs related to the three types of environmental/veterinary surveillance (entomological, ornithological, and equine surveillance), a process analysis was performed identifying all the resources involved in the different actions, in terms of human resources, equipment, and consumables for both on-field and laboratory activities (Table 2).

### 2.2. Blood Transfusion Costs

The screening of blood for transfusion has been one of the most important aspects of the WNV surveillance plan, since 2002, when the first WNV infection due to blood transfusion was detected in the United States [28,29].

Italian national guidelines mandate the screening of blood units between 1 June and 30 November for all blood donations in the provinces in which WNV was detected the previous year [30]. For all other provinces, the controls are activated only after the detection of a WNV-positive specimen (either human or animal—mosquitoes, birds, horses) in the laboratory test with confirmation through NAT [28].

Table 3 reports the number of blood donations tested yearly in Lombardy Region by the regional blood transfusion establishment during the period considered. The NAT cost was derived from the Lombardy Region equivalent, equal to 15.00 EUR [31]

### 2.3. Costs of West Nile Virus Infection in Human Cases

This analysis also considered direct medical costs related to human WNV infection cases confirmed by the Italian National Institute of Health (Istituto Superiore di Sanità—ISS) assuming the Regional Health Service perspective, in terms of inpatient activity and diagnostic activity. Costs were derived from a previously published analysis related to the Italian context [28]. A mean number of 21.3 inpatient days was considered per patient, as reported in the literature [28]. With a conservative approach, the costs considered excluded those related to intensive care units, due to a lack of this information. In Table 4, we report the numbers of annual confirmed human infections and inpatients and horses’ infection cases. The WNV infection in horses, as in humans, can involve neurologic disease and they are incidental hosts to WNV; therefore, they may share common epidemiological aspects regarding disease occurrence and spread. [32].

## 3. Results

The multidisciplinary approach to WNV surveillance made it possible to identify the viral circulation in the study area early. In Table 5 we report the date on which the first positive case was reported by year and by type of surveillance of WNV, together with the date of the first human case of WNV infection detected in each year.

**Figure 1:**
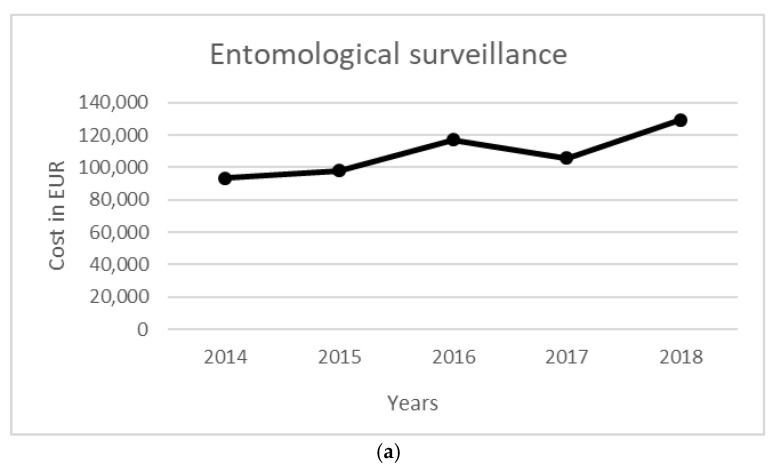

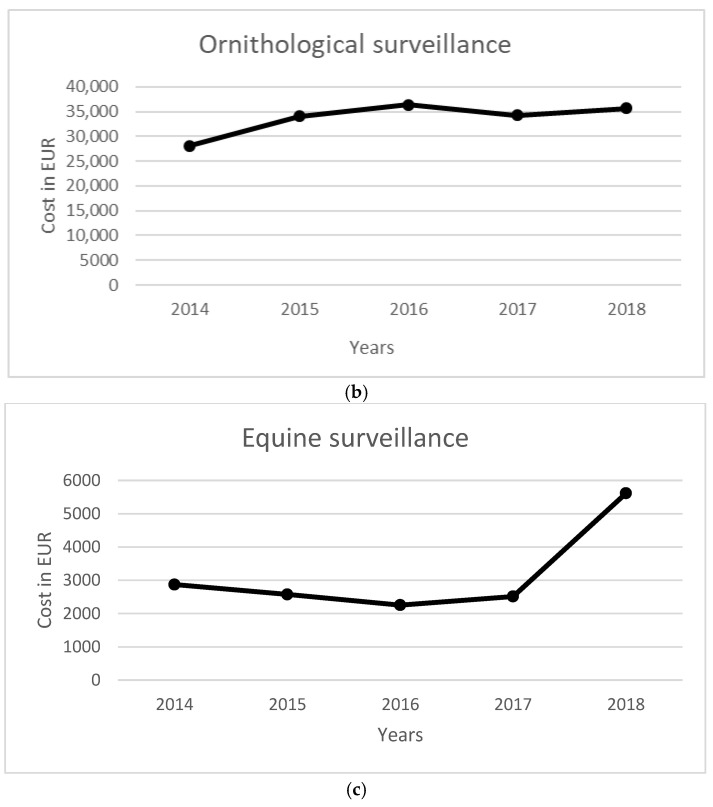
Annual costs per environmental/veterinary surveillance. (**a**) Entomological, (**b**) ornithological, and (**c**) equine surveillance.

**Figure 2:**
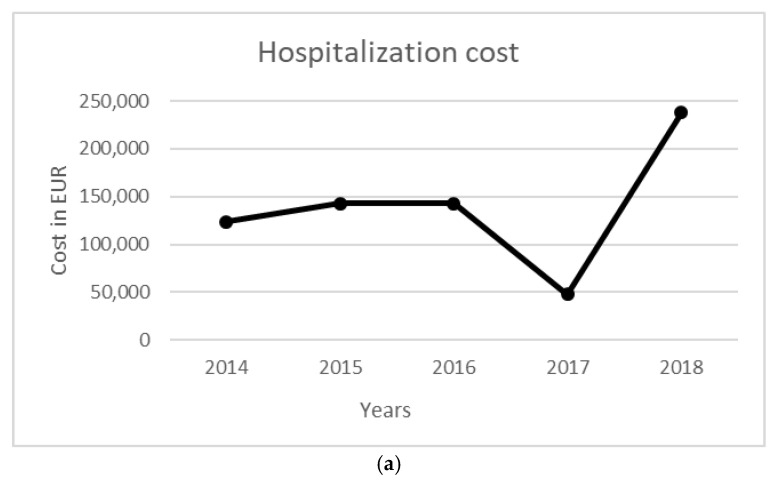

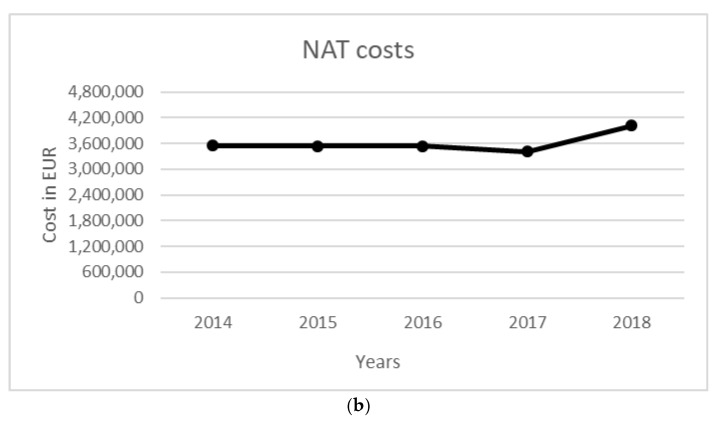
Annual costs per type of human surveillance (hospitalization (**a**) and NAT test (**b**)).

The total cost of the surveillance activity for a five-year period was equal to 542,935 EUR. Costs were mainly related to entomological surveillance (358,574 EUR), followed by ornithological surveillance (168,535 EUR). Total NAT costs were equal to 10.38 million EUR, while the costs to manage WNV-related infections in terms of diagnostic activity and hospitalization were equal to 705,107 EUR. The costs per year for each activity are reported in Table 6, Figure 1 and Figure 2.

**Table 6:** Costs related to surveillance activity, NATs, and human infection management.

	2014	2015	2016	2017	2018	Total
Entomological surveillance (EUR)	64,877	63,869	79,225	68,140	82,463	358,574
Ornithological surveillance (EUR)	28,150	34,077	36,343	34,304	35,661	168,535
Equine surveillance (EUR)	2872	2574	2253	2513	5614	15,826
Total surveillance costs (EUR)	95,899	100,520	117,821	104,957	123,739	542,935
Total NAT costs (EUR)	2,037,015	2,534,130	1,615,305	1,767,180	2,427,255	10,380,885
Infection inpatient management costs (EUR)	124,605	19,170	143,775	47,925	230,040	565,515
Infection diagnostic costs (EUR)	962	148	1110	370	1776	4366
Total costs related to WNV and Usutu infections (EUR)	125,567	19,315	144,885	48,295	231,816	569,881
Total annual costs (EUR)	2,258,481	2,653,968	1,878,011	1,920,432	2,782,809	11,493,701

Considering the number of NATs for checking blood donations, performed starting from the first positive specimen detected by environmental/veterinary surveillance, mean annual avoided costs of 1,535,451 EUR were estimated due to delayed testing on blood units, compared with the scenario in which NATs were performed throughout the vectorial season, i.e., from 1 June to 30 November, as reported in Table 7. The higher estimated savings were related to the years 2016, 2017, and 2018, due to the higher annual number of samples tested as a result of a higher viral circulation.

Overall, considering the five-year period, application of the environmental/veterinary surveillance program allowed a reduction of approximately 7.7 million EUR in costs incurred in Lombardy Region.

## 4. Discussion

Application of the above-described surveillance system allowed the Regional Health and Welfare Unit of Lombardy Region to avoid costs representing more than 40% of the WNV monitoring plan costs. This was due to the possibility to postpone the beginning of the blood samples tests until the first positive specimen detected by environmental/veterinary surveillance.

Moreover, the sharing of information obtained from epidemiological and environmental/veterinary surveillance has enabled controlling the risk of WNV transmission via blood transfusion.

In accordance with White (2001) and Healy et al., (2015) [33,34], the surveillance system adopted was useful in providing important information on viral circulation in the reservoirs, as well as in vectors and the host population. In fact, the results collected from birds and mosquitoes informed the control bodies on the extent of circulation of the virus. Among vertebrate hosts, horses are particularly sensitive to WNV; thus, the detection of infection/disease in such species is highly pertinent and informative from a public health perspective and also gives an estimation of the population of infected vectors [35,36].

Our results also showed that viral activity was generally detected earlier in mosquitoes and birds than in humans [37]. In fact, in the observation period from 2014 to 2018, entomological surveillance revealed the presence of WNV on average 1 month in advance when compared with the detection of the first WNV clinical cases in humans or the detection of positivity in donors, except for 2015, when the first positive case was detected much earlier, i.e., on 21 May in a bird (as reported in Table 5) [38]. The proximity of the dates of the first positive specimen related to the entomological, ornithological, and equine surveillance in 2014 and 2018 may have been due to a higher virus circulation leading to a higher number of WNV infections and human clinical cases. 

It should be noted that the cost of the personnel involved in the human and equine surveillance is not included for laboratory tests, since data available does not allow to isolate the costs of the tests related to WNV. This is due to the fact that the costs available for personnel (payroll) include a sum of activities performed as part of the normal routine activities expected of the specific role (physician or veterinary). Thus, it is not possible to identify the costs related dedicated to the sole aforementioned activities which are part of the surveillance systems.

In this context of West Nile Disease viral circulation control, savings in terms of resources were obtained by not activating NAT screening on blood transfusion or organ donors until circulation of the arbovirus was confirmed at the local (provincial) level. Indeed, studies, in some published cases [24], have demonstrated the sustainability of the system with variable deficit margins.

The costs associated with the management of infected patients vary widely depending on the clinical manifestation of the infection and on the course of the disease, being related to inpatient activity and considering severe cases, long inpatient days, and intensive care unit management.

In Italy, a cost–benefit analysis related to the implementation of the WNV surveillance program in Emilia-Romagna Region estimated 1.21 million EUR of costs saved by avoiding tests for blood donations and up to 2.98 million EUR of costs saved by avoiding hospitalization-related infections between 2009 and 2015 [25]. In this study, Paternoster and colleagues (2017) [25] considered further costs and epidemiological elements when compared to the analysis here presented, such as the lack of transmissions by transfusions and the related costs. Moreover, an additional constraint to the comparison between the results of the two analyses is related to relevant differences in the organization of the surveillance activities in terms of personnel involved in the management of traps. In fact, while in Emilia-Romagna this activity is managed by personnel specifically recruited, in Lombardy Region, it is performed by personnel already in charge at the Veterinary Services.

## 5. Conclusions

An integrated surveillance system, including birds as virus reservoirs, mosquito vectors, and dead-end hosts such as horses and humans, was used to identify the location and timing of viral activity in the Lombardy Region. The information gained through surveillance was used to prevent viral transmission to the human population, as well as to anticipate the detection of WNV in blood and organ donors using NAT [39,40]. At the same time, this plan allowed for the collection of data that could improve understanding of the epidemiology of WNV infection and identify possible risk factors related to human infections.

The above-described surveillance program highlighted the presence of WNV in Lombardy every year in the period 2014–2018, albeit with different intensities, and it allowed the possibility to both document the expansion of the endemic area of WNV in northern Italy and guarantee an avoidance of NAT-related costs. The latter was estimated to be 7.7 million EUR in the five-year period, with mean yearly avoided costs of 1.5 million EUR.

## Figures and Tables

**Table 1:** Cost items included for the estimation of costs of the West Nile Virus (WNV) integrated surveillance system in Lombardy, 2014–2018.

Item	Description	Details
Human surveillance	- Cost of diagnosis of West Nile neurologic disease in suspect cases and hospitalization	Personnel cost not included for laboratory analysis and included for hospitalizations
Entomological surveillance	- Mosquito collection - Screening: PCR on each mosquito pool - Confirmation of each positive pool: PCR, cell culture, and sequencing	Personnel cost included
Ornithological surveillance	- Wild bird collection - Screening: PCR on pool of organs (brain spleen heart and kidney) of individual wild birds	Personnel cost included
Equinesurveillance	- Cost of diagnosis of WNV neurologic disease in suspect horses - Screening: IgM and IgG ELISA, competitive ELISA on serum, PCR on EDTA-blood of alive and dead suspect horses	Personnel costnot included
Blood testing	- Nucleic acid amplification test (NAT) on each blood donor sample	Personnel cost included

**Table 2:** Annual number of specimens and unit cost per analysis (including equipment and human resources for both collection of specimens and laboratory activities).

Year	Entomological Surveillance		Ornithological Surveillance		Equine Surveillance	
	N° Samples	Unit Cost (EUR)	N° Samples	Unit Cost (EUR)	N° Samples	Unit Cost (EUR)
2014	1824	35.57	2638	10.67	898	3.20
2015	1817	35.15	2632	12.95	647	3.98
2016	2276	34.81	2962	12.27	760	2.96
2017	1992	34.21	2752	12.47	426	5.90
2018	2358	34.97	2082	17.13	1333	4.21

**Table 3:** Annual blood donations and NAT performed in Lombardy between 2014 and 2018.

Year	Annual Number of Blood Donations	Number of Tests Performed
2014	236,582	135,801
2015	235,991	168,942
2016	235,991	107,687
2017	227,192	117,812
2018	268,120	161,817
Total	1,203,976	692,059

**Table 4:** Confirmed cases of human and horse West Nile Virus infection.

Year	Confirmed Cases of Human WNV Infection	Confirmed Cases of Horses WNV Infection
2014	13	11
2015	2	5
2016	15	4
2017	2	0
2018	23	10

**Table 5:** First positive cases of West Nile Virus detected by veterinary and human surveillance between 2014 and 2018.

	2014	2015	2016	2017	2018
Entomological surveillance	16 July	7 July	5 July	12 July	3 July
Ornithological surveillance	21 July	20 May	18 August	5 August	6 July
Equine surveillance	18 July	6 August	5 August	5 August	31 July
Donor checking	12 November	6 July	9 August	/	
First human WNV infection case diagnosed	13 August	28 July	1–7 August	1–7 August	1–7 August

**Table 7:** Costs related to surveillance activity NATs.

Year	Differential Number of NATs *	Differential Costs for NATs (EUR)
2014	100,781	1,511,715
2015	67,049	1,005,735
2016	128,304	1,924,560
2017	109,380	1,640,700
2018	106,303	1,594,545
Total	511,817	7,677,255

* Cost difference between the surveillance program scenario and the scenario where NATs were performed for each case throughout the year.

## Data Availability

Not applicable.
